# Methyl 9-(4-methyl­benzene­sulfon­yl)-4-phenyl-9*H*-pyrido[3,4-*b*]indole-3-carboxyl­ate

**DOI:** 10.1107/S2414314626009570

**Published:** 2026-09-18

**Authors:** Heiner Detert, Benjamin Dassonneville, Dieter Schollmeyer

**Affiliations:** a University of Mainz, Department of Chemistry, Duesbergweg 10-14, 55099 Mainz, Germany; Goethe-Universität Frankfurt, Germany

**Keywords:** crystal structure, carboline, heterocycle, sulfonamide

## Abstract

The title β-carboline, C_26_H_20_N_2_O_4_S, which was synthesized *via* 2 + 2+2-cyclo­addition to an alkynylynamide, is presented. There is one mol­ecule in the asymmetric unit. The tricyclic carbolin framework is essentially planar and subtends an angle of 68.43 (19)° with the attached phenyl ring. In the crystal, the mol­ecules are arranged in a zigzag pattern in the *ac* plane. Neighbouring mol­ecules are connected *via* a twofold screw axis or a *C*_2_ axis parallel to the *b* axis or *via* a center of inversion.

## Abstract 2

The crystal structure of a β-carboline, C_26_H_20_N_2_O_4_S, synthesized *via* 2 + 2+2-cyclo­addition to an alkynylynamide, is presented.

## Graphical abstract

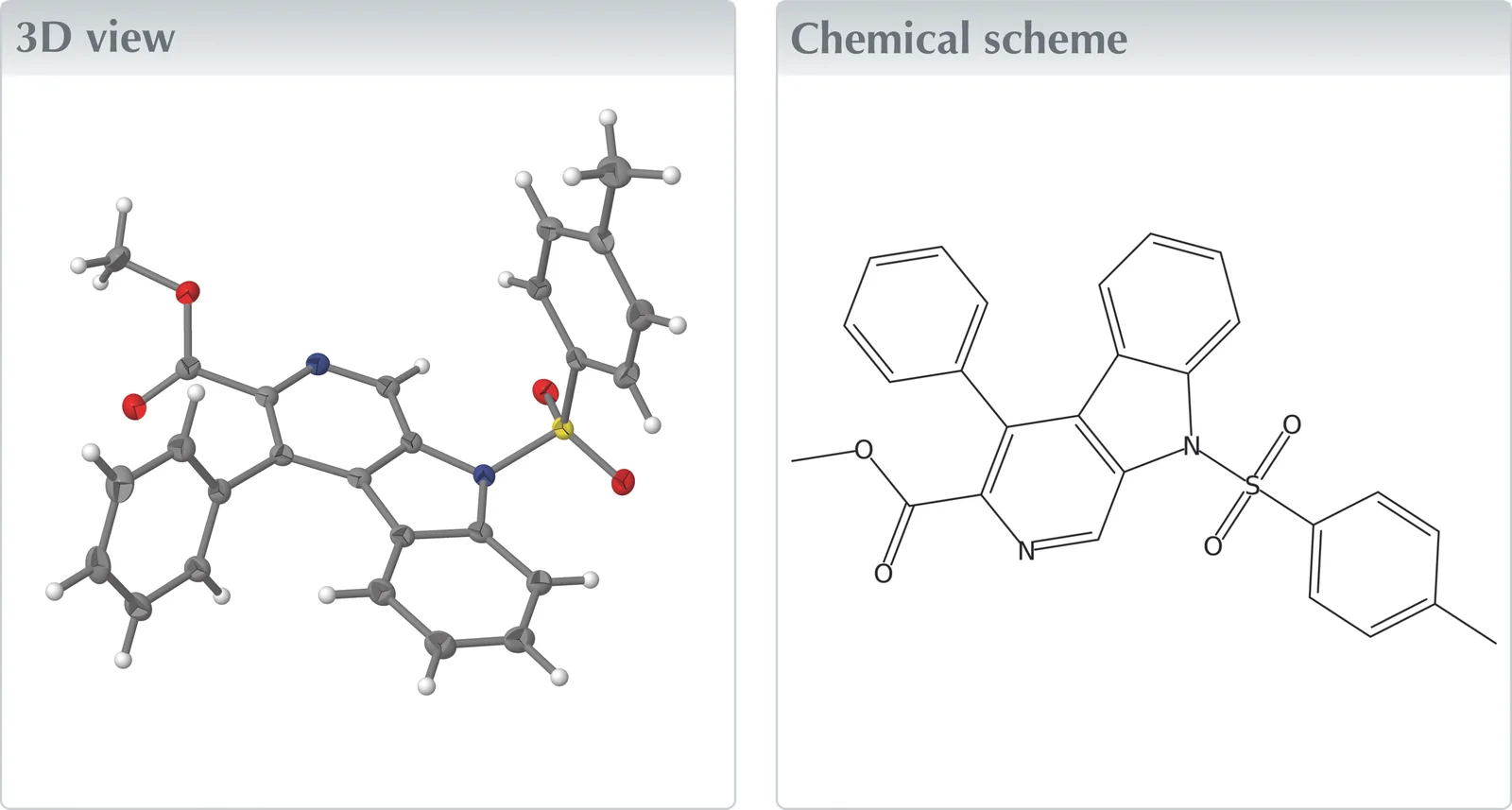

## Structure description

The title compound, C_26_H_20_N_2_O_4_S (Fig. 1 ▸), was prepared in a project on carbolines (Letessier *et al.*, 2012 ▸, 2013 ▸; Letessier & Detert, 2012 ▸; Limbach *et al.*, 2018 ▸) and indolo­thio­pyranes (Dassonneville *et al.*, 2011 ▸, 2023 ▸).

The rhodium-catalyzed 2 + 2 + 2 cyclo­addition of alkynylynamides to nitriles (Nissen & Detert, 2011 ▸) gives two regioisomers, γ-carbolines and, like the title compound, β-carbolines. The tricyclic carbolin framework is essentially planar and subtends an angle of 68.43 (19)° with the attached phenyl ring. The torsion angle of carboline and ester group, O32—C30—C13—N12 amounts to −47.0 (2)° and the planes of carboline and tolyl ring subtend an angle of 83.28 (7)°. Eight mol­ecules of the title compound fill the monoclinic unit cell.

In the crystal, the mol­ecules are arranged in a zigzag pattern in the *ac* plane (Fig. 2 ▸). Neighbouring mol­ecules are connected *via* a twofold screw axis or a *C*_2_ axis parallel to the *b* axis or *via* a center of inversion. Further packing is characterized by alternating phenyl rings and ester groups along the *b*-axis direction.

## Synthesis and crystallization

2-Phenyl­ethyn-1-yl-*N*-tosyl-*N*-ethynylaniline was prepared according to the literature (Dassonneville *et al.*, 2023 ▸). In a dry Schlenk tube were dissolved 7.5 mg of BINAP [2,2′-bis(diphenylphosphino)-1,1′-binaphthyl] and 4.9 mg of Rh(COD)_2_BF_4_ in 3 ml of methyl­ene chloride under argon. The mixture was stirred for 5 min, H_2_ was introduced to activate the catalyst. After stirring for 30 min, the solvent was evaporated and the residue dissolved in 3 ml di­chloro­methane. To this solution was added the diyne (73.4 mg) and methyl cyano­formate (17.3 mg) and the mixture was stirred at 333 K. After the reaction was compete (TLC control), the solvent was removed and the residue was purified to give 61.2 mg of a mixture of the title compound and the γ-isomer. Isolation of the investigated β-carbolin as second fraction after repeated chromatography on silica with petroleum ether/ethyl acetate (9:1), *R*_f_= 0.15. The compound was obtained as a white solid, m.p.= 484–485 K. Assignment of NMR signals follows IUPAC nomenclature and is based on two-dimensional NMR experiments. ^1^H-NMR (400 MHz, CDCl_3_, 298 K): δ = 9.74 (*s*, 1 H, 1-H), 8.38 (*d*, ^3^*J*_H,H_ = 8.4 Hz, 1 H, 8-H), 7.82 (*d*, ^3^*J*_H,H_ = 8.2 Hz, 2 H, 2-H, 6 H, tos), 7.53 (*m*, 4 H, 7-H, 3′-H, 4′-H, 5′-H), 7.17 (*d*, ^3^*J*_H,H_ = 7.6 Hz, 2 H, 2′-H, 6′-H), 7.10 (*t*, ^3^*J*_H,H_ = 7.4 Hz, 1 H, 6-H), 6.67 (*d*, ^3^*J*_H,H_ = 8.0 Hz, 1H, 6-H), 3.78 (*s*, 3 H, OCH_3_), 2.33 (*s*, 3 H, CH_3_); ^13^C-NMR (CDCl_3_): δ = 166.2 (C_q_ C-10), 145.9 (C_q_ C-4 tos), 139.2 C_q_ C-8a); 136.0 C_q_ C-4), 135.5 (CH, C-1), 135.3 (C_q_ C-9a), 134.4 (C_q_, C-1 tos), 133.0 (C_q_ C-4a), 131.8 (C_q_ C-1′), 130.1 (CH, C-3, C-5 tos), 130.0 (CH, C-7), 128.8 & 128.3 (CH, C-2′, C-3′, C-5′, C-6′), 128.5 (CH, C-4 tos), 126.7 (CH, C-2, C-5 tos), 124.2 (CHH, C-6), 124.1 (C_q_, C-4 b), 123.7 (CH, C-5), 114.7 (CH, C-8), 52.6 (O—CH_3_), 21.6 (CH_3_ tos); IR (neat, ATR): 3005*w*, 1736*vs*, 1587*w*, 1421*w*, 1375*vs*, 1254*s*, 1173*vs*, 972*s*, 817*w*, 734*vs*, 696*s*, 661*s* cm^−1^. FD–MS: *m*/*z* (%) = 4455.6 (100). [*M*]^+.^ C_26_H_20_N_2_O_4_S (461.13) calculated: C 68.41, H 4.42, N 6.14, S 7.02; found: C 68.27, H 4.42, N 6.12, S 7.03. Single crystals were grown by slow evaporation of a solution in ethyl acetate

## Refinement

Crystal data, data collection and structure refinement details are summarized in Table 1 ▸.

## Supplementary Material

Crystal structure: contains datablock(s) I, global. DOI: 10.1107/S2414314626009570/bt4207sup1.cif

Structure factors: contains datablock(s) I. DOI: 10.1107/S2414314626009570/bt4207Isup2.hkl

Supporting information file. DOI: 10.1107/S2414314626009570/bt4207Isup3.cml

CCDC reference: 2587814

Additional supporting information:  crystallographic information; 3D view; checkCIF report

## Figures and Tables

**Figure 1:**
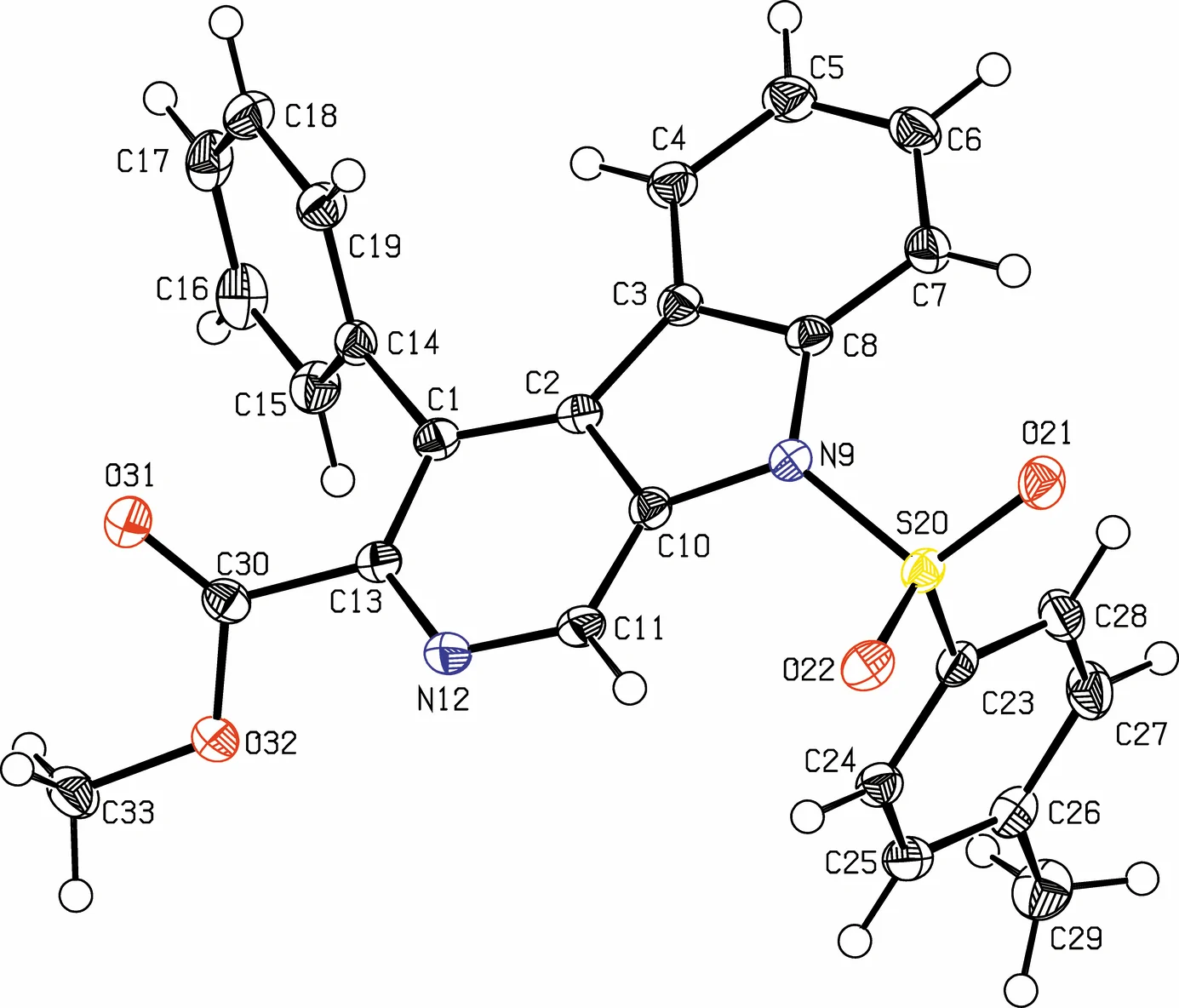
View of the title compound. Displacement ellipsoids are drawn at the 50% probability level (Spek, 2009 ▸).

**Figure 2:**
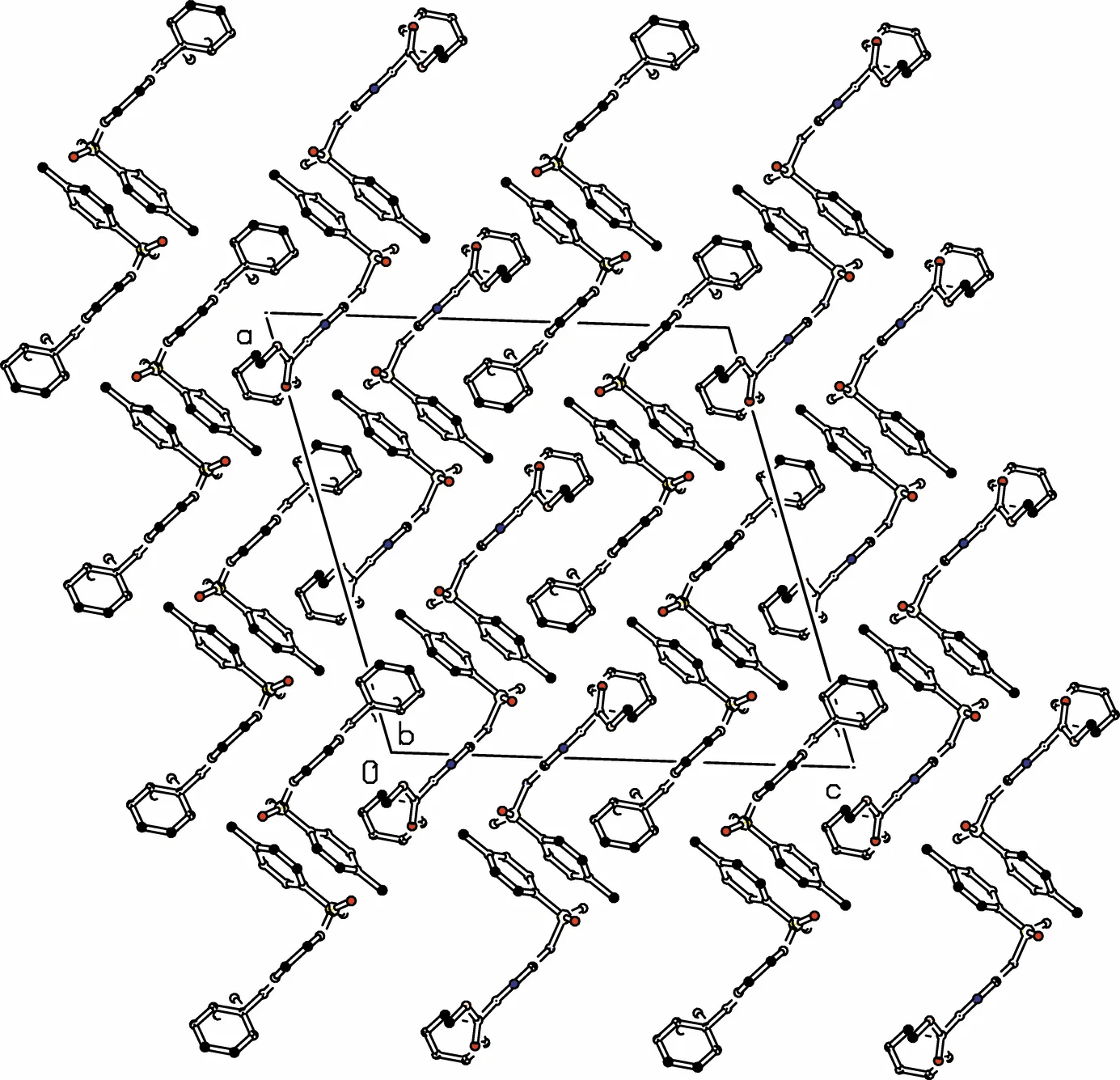
Part of the packing diagram. View along *b*-axis direction (Spek, 2009 ▸). Hydrogen atoms removed for clarity.

**Table 1:** Experimental details

Crystal data	
Chemical formula	C_26_H_20_N_2_O_4_S
*M*_r_	456.50
Crystal system, space group	Monoclinic, *C*2/*c*
Temperature (K)	120
*a*, *b*, *c* (Å)	21.2155 (10), 9.9487 (3), 21.5194 (9)
β (°)	107.842 (3)
*V* (Å^3^)	4323.6 (3)
*Z*	8
Radiation type	Mo *K*α
μ (mm^−1^)	0.19
Crystal size (mm)	0.21 × 0.21 × 0.12

Data collection	
Diffractometer	Stoe *IPDS* 2T
No. of measured, independent and observed [*I* > 2σ(*I*)] reflections	9252, 5086, 4095
*R*_int_	0.032
(sin θ/λ)_max_ (Å^−1^)	0.658

Refinement	
*R*[*F*^2^ > 2σ(*F*^2^)], *wR*(*F*^2^), *S*	0.047, 0.112, 1.07
No. of reflections	5086
No. of parameters	300
H-atom treatment	H-atom parameters constrained
Δρ_max_, Δρ_min_ (e Å^−3^)	0.43, −0.42

Computer programs: *X-AREA WinXpose*, *X-AREA Recipe* and *X-AREA Integrate* (Stoe & Cie, 2020 ▸), *SHELXT2014* (Sheldrick, 2015*a* ▸), *SHELXL2019/2* (Sheldrick, 2015*b* ▸) and *PLATON* (Spek, 2009 ▸).

## full crystallographic data

### Methyl 9-(4-methylbenzenesulfonyl)-4-phenyl-9H-pyrido[3,4-b]indole-3-carboxylate Crystal data

**Table:**

C_26_H_20_N_2_O_4_S	*F*(000) = 1904
*M**_r_* = 456.50	*D*_x_ = 1.403 Mg m^−^^3^
Monoclinic, *C*2/*c*	Mo *K*α radiation, λ = 0.71073 Å
*a* = 21.2155 (10) Å	Cell parameters from 9973 reflections
*b* = 9.9487 (3) Å	θ = 3.2–28.4°
*c* = 21.5194 (9) Å	µ = 0.19 mm^−^^1^
β = 107.842 (3)°	*T* = 120 K
*V* = 4323.6 (3) Å^3^	Block, colorless
*Z* = 8	0.21 × 0.21 × 0.12 mm

### Methyl 9-(4-methylbenzenesulfonyl)-4-phenyl-9H-pyrido[3,4-b]indole-3-carboxylate Data collection

**Table:**

Stoe IPDS 2T diffractometer	*R*_int_ = 0.032
Detector resolution: 6.67 pixels mm^-1^	θ_max_ = 27.9°, θ_min_ = 3.2°
rotation method, ω scans	*h* = −27→21
9252 measured reflections	*k* = −11→13
5086 independent reflections	*l* = −28→28
4095 reflections with *I* > 2σ(*I*)	

### Methyl 9-(4-methylbenzenesulfonyl)-4-phenyl-9H-pyrido[3,4-b]indole-3-carboxylate Refinement

**Table:**

Refinement on *F*^2^	Primary atom site location: dual
Least-squares matrix: full	Hydrogen site location: inferred from neighbouring sites
*R*[*F*^2^ > 2σ(*F*^2^)] = 0.047	H-atom parameters constrained
*wR*(*F*^2^) = 0.112	*w* = 1/[σ^2^(*F*_o_^2^) + (0.0367*P*)^2^ + 9.0512*P*] where *P* = (*F*_o_^2^ + 2*F*_c_^2^)/3
*S* = 1.07	(Δ/σ)_max_ = 0.001
5086 reflections	Δρ_max_ = 0.43 e Å^−^^3^
300 parameters	Δρ_min_ = −0.42 e Å^−^^3^
0 restraints	

### Methyl 9-(4-methylbenzenesulfonyl)-4-phenyl-9H-pyrido[3,4-b]indole-3-carboxylate Special details

**Table:**

Geometry. All esds (except the esd in the dihedral angle between two l.s. planes) are estimated using the full covariance matrix. The cell esds are taken into account individually in the estimation of esds in distances, angles and torsion angles; correlations between esds in cell parameters are only used when they are defined by crystal symmetry. An approximate (isotropic) treatment of cell esds is used for estimating esds involving l.s. planes.
Refinement. Hydrogen atoms were placed at calculated positions and were refined in the riding-model approximation with C_aromatic_–H = 0.95 Å and with *U*_iso_(H) = 1.2 *U*_eq_(C) or C_methyl_–H = 0.98 Å and with *U*_iso_(H) = 1.5 *U*_eq_(C). The methyl groups were allowed to rotate but not to tip.

### Methyl 9-(4-methylbenzenesulfonyl)-4-phenyl-9H-pyrido[3,4-b]indole-3-carboxylate Fractional atomic coordinates and isotropic or equivalent isotropic displacement parameters (Å^2^)

**Table:**

	*x*	*y*	*z*	*U*_iso_*/*U*_eq_	
C1	0.43628 (9)	0.63823 (19)	0.57067 (8)	0.0176 (4)	
C2	0.48132 (9)	0.70460 (19)	0.62341 (9)	0.0168 (4)	
C3	0.49768 (9)	0.84527 (19)	0.63877 (9)	0.0180 (4)	
C4	0.47465 (10)	0.9659 (2)	0.60650 (10)	0.0223 (4)	
H4	0.441858	0.965309	0.564946	0.027*	
C5	0.50013 (10)	1.0859 (2)	0.63573 (10)	0.0252 (4)	
H5	0.484592	1.168352	0.614199	0.030*	
C6	0.54853 (10)	1.0872 (2)	0.69666 (10)	0.0254 (4)	
H6	0.564912	1.171112	0.716002	0.030*	
C7	0.57338 (10)	0.9699 (2)	0.72976 (10)	0.0218 (4)	
H7	0.606510	0.971564	0.771097	0.026*	
C8	0.54752 (9)	0.84933 (19)	0.69960 (9)	0.0180 (4)	
N9	0.56181 (7)	0.71532 (16)	0.72326 (7)	0.0174 (3)	
C10	0.52207 (9)	0.62805 (19)	0.67500 (8)	0.0172 (4)	
C11	0.51848 (9)	0.48836 (19)	0.67375 (9)	0.0202 (4)	
H11	0.545559	0.437924	0.709489	0.024*	
N12	0.47753 (8)	0.42496 (16)	0.62301 (8)	0.0204 (3)	
C13	0.43767 (9)	0.49812 (19)	0.57382 (9)	0.0188 (4)	
C14	0.39167 (9)	0.71401 (19)	0.51435 (9)	0.0179 (4)	
C15	0.40138 (10)	0.7085 (2)	0.45318 (9)	0.0236 (4)	
H15	0.436108	0.655121	0.447024	0.028*	
C16	0.36047 (10)	0.7809 (2)	0.40129 (9)	0.0277 (4)	
H16	0.367077	0.776250	0.359628	0.033*	
C17	0.31007 (10)	0.8597 (2)	0.40996 (10)	0.0268 (4)	
H17	0.282492	0.909921	0.374423	0.032*	
C18	0.29982 (10)	0.8654 (2)	0.47059 (10)	0.0253 (4)	
H18	0.265057	0.918961	0.476547	0.030*	
C19	0.34049 (9)	0.7926 (2)	0.52250 (9)	0.0221 (4)	
H19	0.333341	0.796404	0.563933	0.027*	
S20	0.63546 (2)	0.66836 (5)	0.77386 (2)	0.01780 (11)	
O21	0.65626 (7)	0.77486 (15)	0.82004 (6)	0.0240 (3)	
O22	0.62633 (7)	0.53544 (15)	0.79476 (6)	0.0227 (3)	
C23	0.68821 (9)	0.6622 (2)	0.72515 (8)	0.0181 (4)	
C24	0.69120 (9)	0.5463 (2)	0.68989 (9)	0.0196 (4)	
H24	0.664623	0.470560	0.691979	0.024*	
C25	0.73339 (9)	0.5422 (2)	0.65162 (9)	0.0213 (4)	
H25	0.735583	0.463408	0.627426	0.026*	
C26	0.77254 (9)	0.6532 (2)	0.64856 (9)	0.0225 (4)	
C27	0.76843 (10)	0.7683 (2)	0.68399 (10)	0.0264 (4)	
H27	0.794930	0.844264	0.682083	0.032*	
C28	0.72618 (10)	0.7737 (2)	0.72203 (9)	0.0243 (4)	
H28	0.723345	0.852955	0.745653	0.029*	
C29	0.81714 (11)	0.6503 (3)	0.60604 (10)	0.0311 (5)	
H29A	0.832160	0.557958	0.603197	0.047*	
H29B	0.855549	0.708319	0.624977	0.047*	
H29C	0.792749	0.682566	0.562243	0.047*	
C30	0.39117 (9)	0.41361 (19)	0.52160 (9)	0.0194 (4)	
O31	0.33274 (7)	0.43197 (15)	0.49932 (7)	0.0267 (3)	
O32	0.42403 (7)	0.31138 (15)	0.50485 (7)	0.0258 (3)	
C33	0.38414 (11)	0.2172 (2)	0.45801 (10)	0.0274 (4)	
H33A	0.365635	0.262088	0.415801	0.041*	
H33B	0.348011	0.184149	0.473394	0.041*	
H33C	0.411710	0.141355	0.453070	0.041*	

### Methyl 9-(4-methylbenzenesulfonyl)-4-phenyl-9H-pyrido[3,4-b]indole-3-carboxylate Atomic displacement parameters (Å^2^)

**Table:**

	*U*^11^	*U*^22^	*U*^33^	*U*^12^	*U*^13^	*U*^23^
C1	0.0174 (8)	0.0187 (9)	0.0173 (8)	0.0021 (7)	0.0063 (7)	0.0008 (7)
C2	0.0165 (8)	0.0156 (9)	0.0190 (8)	0.0023 (7)	0.0067 (7)	0.0012 (7)
C3	0.0161 (8)	0.0180 (9)	0.0211 (8)	0.0003 (7)	0.0076 (7)	−0.0006 (7)
C4	0.0203 (9)	0.0197 (9)	0.0262 (9)	0.0014 (8)	0.0061 (7)	0.0031 (8)
C5	0.0235 (10)	0.0167 (10)	0.0356 (11)	0.0005 (8)	0.0096 (8)	0.0038 (8)
C6	0.0255 (10)	0.0172 (10)	0.0355 (11)	−0.0031 (8)	0.0123 (9)	−0.0034 (8)
C7	0.0193 (9)	0.0204 (10)	0.0260 (9)	−0.0019 (7)	0.0075 (7)	−0.0032 (8)
C8	0.0165 (8)	0.0178 (9)	0.0209 (8)	0.0020 (7)	0.0078 (7)	0.0006 (7)
N9	0.0160 (7)	0.0171 (8)	0.0180 (7)	0.0012 (6)	0.0036 (6)	0.0002 (6)
C10	0.0153 (8)	0.0191 (9)	0.0172 (8)	0.0009 (7)	0.0050 (7)	−0.0011 (7)
C11	0.0203 (9)	0.0177 (9)	0.0206 (9)	0.0046 (7)	0.0032 (7)	0.0024 (7)
N12	0.0209 (8)	0.0170 (8)	0.0217 (8)	0.0026 (6)	0.0041 (6)	−0.0004 (6)
C13	0.0175 (8)	0.0183 (9)	0.0200 (8)	0.0026 (7)	0.0050 (7)	−0.0012 (7)
C14	0.0179 (8)	0.0156 (9)	0.0190 (8)	−0.0017 (7)	0.0039 (7)	0.0011 (7)
C15	0.0216 (9)	0.0275 (11)	0.0230 (9)	−0.0004 (8)	0.0086 (7)	−0.0009 (8)
C16	0.0291 (11)	0.0346 (12)	0.0188 (9)	−0.0066 (9)	0.0063 (8)	0.0027 (8)
C17	0.0259 (10)	0.0252 (11)	0.0222 (9)	−0.0034 (8)	−0.0030 (8)	0.0088 (8)
C18	0.0211 (9)	0.0230 (10)	0.0288 (10)	0.0032 (8)	0.0032 (8)	0.0029 (8)
C19	0.0214 (9)	0.0232 (10)	0.0212 (9)	0.0019 (8)	0.0058 (7)	0.0005 (7)
S20	0.0159 (2)	0.0217 (2)	0.01493 (19)	0.00209 (17)	0.00344 (15)	−0.00003 (17)
O21	0.0219 (7)	0.0308 (8)	0.0178 (6)	0.0007 (6)	0.0040 (5)	−0.0048 (6)
O22	0.0220 (7)	0.0255 (8)	0.0208 (6)	0.0029 (6)	0.0071 (5)	0.0052 (6)
C23	0.0141 (8)	0.0229 (9)	0.0159 (8)	0.0018 (7)	0.0026 (6)	0.0000 (7)
C24	0.0186 (8)	0.0184 (9)	0.0215 (8)	0.0013 (7)	0.0057 (7)	0.0020 (7)
C25	0.0227 (9)	0.0215 (10)	0.0195 (8)	0.0042 (8)	0.0060 (7)	−0.0001 (7)
C26	0.0176 (9)	0.0292 (11)	0.0200 (8)	0.0019 (8)	0.0048 (7)	0.0012 (8)
C27	0.0223 (10)	0.0274 (11)	0.0303 (10)	−0.0078 (8)	0.0091 (8)	−0.0047 (8)
C28	0.0236 (9)	0.0236 (10)	0.0256 (9)	−0.0043 (8)	0.0074 (8)	−0.0073 (8)
C29	0.0272 (10)	0.0407 (13)	0.0299 (10)	0.0004 (10)	0.0155 (9)	−0.0013 (9)
C30	0.0232 (9)	0.0159 (9)	0.0195 (8)	−0.0001 (7)	0.0071 (7)	0.0013 (7)
O31	0.0205 (7)	0.0286 (8)	0.0278 (7)	0.0013 (6)	0.0029 (6)	−0.0053 (6)
O32	0.0236 (7)	0.0238 (8)	0.0265 (7)	0.0032 (6)	0.0023 (6)	−0.0087 (6)
C33	0.0331 (11)	0.0234 (10)	0.0235 (9)	0.0004 (9)	0.0053 (8)	−0.0068 (8)

### Methyl 9-(4-methylbenzenesulfonyl)-4-phenyl-9H-pyrido[3,4-b]indole-3-carboxylate Geometric parameters (Å, º)

**Table:**

C1—C13	1.395 (3)	C17—C18	1.388 (3)
C1—C2	1.404 (3)	C17—H17	0.9500
C1—C14	1.493 (2)	C18—C19	1.388 (3)
C2—C10	1.404 (2)	C18—H18	0.9500
C2—C3	1.455 (3)	C19—H19	0.9500
C3—C4	1.398 (3)	S20—O21	1.4270 (15)
C3—C8	1.409 (3)	S20—O22	1.4290 (15)
C4—C5	1.380 (3)	S20—C23	1.7532 (18)
C4—H4	0.9500	C23—C28	1.385 (3)
C5—C6	1.396 (3)	C23—C24	1.393 (3)
C5—H5	0.9500	C24—C25	1.390 (3)
C6—C7	1.384 (3)	C24—H24	0.9500
C6—H6	0.9500	C25—C26	1.396 (3)
C7—C8	1.394 (3)	C25—H25	0.9500
C7—H7	0.9500	C26—C27	1.394 (3)
C8—N9	1.426 (2)	C26—C29	1.505 (3)
N9—C10	1.416 (2)	C27—C28	1.388 (3)
N9—S20	1.6741 (16)	C27—H27	0.9500
C10—C11	1.392 (3)	C28—H28	0.9500
C11—N12	1.328 (2)	C29—H29A	0.9800
C11—H11	0.9500	C29—H29B	0.9800
N12—C13	1.348 (2)	C29—H29C	0.9800
C13—C30	1.504 (3)	C30—O31	1.198 (2)
C14—C19	1.392 (3)	C30—O32	1.343 (2)
C14—C15	1.394 (3)	O32—C33	1.445 (2)
C15—C16	1.387 (3)	C33—H33A	0.9800
C15—H15	0.9500	C33—H33B	0.9800
C16—C17	1.384 (3)	C33—H33C	0.9800
C16—H16	0.9500		
			
C13—C1—C2	115.56 (17)	C18—C17—H17	120.0
C13—C1—C14	122.85 (17)	C19—C18—C17	119.84 (19)
C2—C1—C14	121.57 (17)	C19—C18—H18	120.1
C10—C2—C1	119.05 (17)	C17—C18—H18	120.1
C10—C2—C3	107.20 (16)	C18—C19—C14	120.55 (18)
C1—C2—C3	133.75 (17)	C18—C19—H19	119.7
C4—C3—C8	119.05 (18)	C14—C19—H19	119.7
C4—C3—C2	133.65 (17)	O21—S20—O22	120.71 (8)
C8—C3—C2	107.30 (16)	O21—S20—N9	105.88 (8)
C5—C4—C3	119.20 (18)	O22—S20—N9	105.87 (8)
C5—C4—H4	120.4	O21—S20—C23	108.94 (9)
C3—C4—H4	120.4	O22—S20—C23	109.19 (9)
C4—C5—C6	120.57 (19)	N9—S20—C23	105.10 (8)
C4—C5—H5	119.7	C28—C23—C24	120.85 (17)
C6—C5—H5	119.7	C28—C23—S20	119.24 (15)
C7—C6—C5	122.02 (19)	C24—C23—S20	119.91 (15)
C7—C6—H6	119.0	C25—C24—C23	119.46 (18)
C5—C6—H6	119.0	C25—C24—H24	120.3
C6—C7—C8	116.89 (18)	C23—C24—H24	120.3
C6—C7—H7	121.6	C24—C25—C26	120.36 (18)
C8—C7—H7	121.6	C24—C25—H25	119.8
C7—C8—C3	122.24 (18)	C26—C25—H25	119.8
C7—C8—N9	128.91 (17)	C27—C26—C25	119.13 (18)
C3—C8—N9	108.77 (16)	C27—C26—C29	120.34 (19)
C10—N9—C8	107.32 (14)	C25—C26—C29	120.50 (19)
C10—N9—S20	122.20 (13)	C28—C27—C26	120.95 (19)
C8—N9—S20	123.16 (13)	C28—C27—H27	119.5
C11—C10—C2	120.63 (17)	C26—C27—H27	119.5
C11—C10—N9	130.02 (17)	C23—C28—C27	119.23 (19)
C2—C10—N9	109.34 (16)	C23—C28—H28	120.4
N12—C11—C10	120.56 (17)	C27—C28—H28	120.4
N12—C11—H11	119.7	C26—C29—H29A	109.5
C10—C11—H11	119.7	C26—C29—H29B	109.5
C11—N12—C13	118.96 (17)	H29A—C29—H29B	109.5
N12—C13—C1	125.19 (17)	C26—C29—H29C	109.5
N12—C13—C30	113.26 (17)	H29A—C29—H29C	109.5
C1—C13—C30	121.51 (17)	H29B—C29—H29C	109.5
C19—C14—C15	119.17 (18)	O31—C30—O32	124.50 (18)
C19—C14—C1	120.41 (16)	O31—C30—C13	125.10 (18)
C15—C14—C1	120.42 (17)	O32—C30—C13	110.37 (16)
C16—C15—C14	120.18 (19)	C30—O32—C33	115.91 (16)
C16—C15—H15	119.9	O32—C33—H33A	109.5
C14—C15—H15	119.9	O32—C33—H33B	109.5
C17—C16—C15	120.28 (18)	H33A—C33—H33B	109.5
C17—C16—H16	119.9	O32—C33—H33C	109.5
C15—C16—H16	119.9	H33A—C33—H33C	109.5
C16—C17—C18	119.98 (18)	H33B—C33—H33C	109.5
C16—C17—H17	120.0		
			
C13—C1—C2—C10	−1.8 (2)	C13—C1—C14—C19	113.3 (2)
C14—C1—C2—C10	−179.94 (16)	C2—C1—C14—C19	−68.7 (2)
C13—C1—C2—C3	177.82 (19)	C13—C1—C14—C15	−67.7 (3)
C14—C1—C2—C3	−0.4 (3)	C2—C1—C14—C15	110.3 (2)
C10—C2—C3—C4	179.38 (19)	C19—C14—C15—C16	0.1 (3)
C1—C2—C3—C4	−0.2 (4)	C1—C14—C15—C16	−178.95 (19)
C10—C2—C3—C8	−0.5 (2)	C14—C15—C16—C17	0.5 (3)
C1—C2—C3—C8	179.86 (19)	C15—C16—C17—C18	−0.7 (3)
C8—C3—C4—C5	−1.6 (3)	C16—C17—C18—C19	0.4 (3)
C2—C3—C4—C5	178.54 (19)	C17—C18—C19—C14	0.2 (3)
C3—C4—C5—C6	0.3 (3)	C15—C14—C19—C18	−0.4 (3)
C4—C5—C6—C7	0.7 (3)	C1—C14—C19—C18	178.63 (18)
C5—C6—C7—C8	−0.4 (3)	C10—N9—S20—O21	−172.82 (14)
C6—C7—C8—C3	−1.0 (3)	C8—N9—S20—O21	40.91 (16)
C6—C7—C8—N9	−177.29 (18)	C10—N9—S20—O22	−43.55 (16)
C4—C3—C8—C7	2.0 (3)	C8—N9—S20—O22	170.18 (13)
C2—C3—C8—C7	−178.12 (16)	C10—N9—S20—C23	71.95 (16)
C4—C3—C8—N9	178.94 (16)	C8—N9—S20—C23	−74.32 (15)
C2—C3—C8—N9	−1.15 (19)	O21—S20—C23—C28	−19.10 (18)
C7—C8—N9—C10	179.08 (18)	O22—S20—C23—C28	−152.82 (15)
C3—C8—N9—C10	2.36 (19)	N9—S20—C23—C28	93.99 (16)
C7—C8—N9—S20	−30.4 (3)	O21—S20—C23—C24	161.28 (14)
C3—C8—N9—S20	152.88 (13)	O22—S20—C23—C24	27.56 (17)
C1—C2—C10—C11	0.6 (3)	N9—S20—C23—C24	−85.63 (16)
C3—C2—C10—C11	−179.05 (17)	C28—C23—C24—C25	0.6 (3)
C1—C2—C10—N9	−178.31 (15)	S20—C23—C24—C25	−179.74 (14)
C3—C2—C10—N9	2.00 (19)	C23—C24—C25—C26	0.1 (3)
C8—N9—C10—C11	178.48 (19)	C24—C25—C26—C27	−0.4 (3)
S20—N9—C10—C11	27.6 (3)	C24—C25—C26—C29	−178.65 (18)
C8—N9—C10—C2	−2.71 (19)	C25—C26—C27—C28	0.0 (3)
S20—N9—C10—C2	−153.57 (13)	C29—C26—C27—C28	178.26 (19)
C2—C10—C11—N12	1.6 (3)	C24—C23—C28—C27	−1.0 (3)
N9—C10—C11—N12	−179.73 (17)	S20—C23—C28—C27	179.36 (16)
C10—C11—N12—C13	−2.5 (3)	C26—C27—C28—C23	0.7 (3)
C11—N12—C13—C1	1.2 (3)	N12—C13—C30—O31	131.0 (2)
C11—N12—C13—C30	−176.58 (16)	C1—C13—C30—O31	−46.9 (3)
C2—C1—C13—N12	0.9 (3)	N12—C13—C30—O32	−47.0 (2)
C14—C1—C13—N12	179.06 (16)	C1—C13—C30—O32	135.12 (18)
C2—C1—C13—C30	178.56 (16)	O31—C30—O32—C33	−1.9 (3)
C14—C1—C13—C30	−3.3 (3)	C13—C30—O32—C33	176.09 (16)
